# Advancements in Sports Medicine

**DOI:** 10.3390/jcm12103489

**Published:** 2023-05-16

**Authors:** Yaying Sun, Jiwu Chen

**Affiliations:** 1 Huashan Hospital, Fudan University, Shanghai 200040, China; 2 Department of Sports Medicine, Shanghai General Hospital, Shanghai Jiaotong University, Shanghai 200080, China

Sports medicine has developed rapidly in recent years. Countless advancements have been achieved regarding the mechanism, repair, and recovery of sports injuries. The interaction between sports medicine and other disciplines is also a trending topic. Multi-disciplinary outcomes are being increasingly yielded. In this Special Issue, we have collected several clinical advancements in sports medicine.

Rotator cuff tear is a common shoulder disorder in clinical practice. Based on tear size, treatment methods can be divided into direct repair using suture anchors or repair with augmentation due to a massive tear size. Li et al. conducted a review of the current literature and reported that bone quality, insertion depth, insertion angle, size of rotator cuff tear, preoperative corticosteroid injections, anchor design, and the materials used to produce anchors may influence the anchor pullout strength, leading to a poor recovery [1]. Regarding massive rotator cuff tear that cannot be fixed directly, Wellington et al. used a biologically enhanced demineralized bone matrix for the augmentation and found that 10 of 20 patients who received this treatment still suffered from a retear at follow-up [2]. This outcome suggested that there is still a long way to go to enhance the repair of rotator cuff.

For trunk, spinal fusion is usually applied for patients with lumbar degeneration with overall good results. However, some may still experience failure. Guo et al. noticed that the albumin-to-alkaline phosphatase ratio can be used as a prognostic biomarker for measuring clinical outcomes after spinal fusion [3]. Regarding lower limb, Cong et al. reported a modified capsulotomy approach to facilitate the arthroscopic femoroplasty and acetabular labrum repair, with the clinical data supporting its popularization [4]. Regarding knee, Zhang et al. found that anterior cruciate ligament reconstruction using an insertion preservation technique has a protective effect on cartilage degeneration in long-term follow-up [5].

In addition to the general population, novel advancements have also been achieved regarding injuries in athletes. Martins established a predictive model for injury risk in football players [6], while Keller et al. pointed out some divergences in terms of exaggerated blood pressure response in athletes [7]. Gaudette et al. studied runner injuries and emphasized the importance of gait retaining for post-injury recovery [8]. Merle et al. focused on the oral health of young athletes and revealed an association of blood/performance indexes and periodontal inflammation [9].

Some interesting multi-disciplinary research is also included in this Issue. Guo et al. conducted a systematic review and found that aerobic plus machine-assisted resistance training may improve the vascular function in patients with type 2 diabetes [10], while Ma et al. found that adaptive posture-balance cardiac rehabilitation exercise could remarkably restore physical tolerance in a population suffering from cardiovascular diseases [11]. The interaction between sports medicine and other subjects will no doubt make this discipline more meaningful in the future.
